# Case in Which a Woman Was Successfully Tapped in the Sixth Month of Pregnancy

**Published:** 1827-05

**Authors:** James Russell

**Affiliations:** Surgeon.


### ASCITES WITH PREGNANCY.

*Case in which a Woman was successfully Tapped in the sixth Month of Pregnancy.* By James Russell, Esq. Surgeon.

On the 26th of May, 1826, I visited Mrs. Kelly, a poor woman, labouring under ascites and general debility. According to her own account, she had advanced to the sixth month of utero gestation. The abdomen was distended to a very considerable size, and the legs were anasarcous; the rest of the body was much emaciated. Dyspnœa and constipation of the bowels were the symptoms which demanded relief;—I bled her, and prescribed a dose of Calomel and Jalap.

27th.—To-day, the symptoms are relieved, and she says that she feels tolerably easy.

June 8th.—My attendance has not been called for since the last report, until to-day. She now labours under dyspnœa, with pain and tension of the abdomen.

A vein was opened, and the blood allowed to flow till ease was procured. A dose of Calomel and Jalap was prescribed, and desired to be repeated thrice a-week.

19th.—Nothing worthy of remark has occurred since the last report. The dyspnœa and cough at present, however, are of so alarming a character, as to induce me to decide on tapping her.

20th.—The dyspnœa and cough are very severe; the pulse is quick and hard; the urine is scanty and high coloured; the labia pudendi are very much distended with serum. Her uneasiness is extreme, and she refers it to the extraordinary distention of the abdominal integuments. Her period of pregnancy is now nearly seven months. I introduced the trocar two inches below the umbilicus. When about eight quarts of the fluid had escaped, she manifested so much uneasiness as to induce me to check any further discharge, and to allow her to lie down. She recovered in a few minutes. Twenty-three quarts of fluid were drawn off. Several times during the operation, the tendency to syncope rendered it necessary for her to assume the horizontal posture.

Nine P.M.—Respiration easy; no cough; pulse ninety-four. She says that, two hours after the operation, she passed two pints of limpid urine.

21st, eleven A.M.—I found her sitting up in bed: she expresses herself to be considerably relieved. The tumefaction of the labia pudendi has subsided, and that of the legs is also much less. The respiration is natural. She made water four times in the course of the night, and not less than a pint each time. From eight o'clock this morning to the present hour, she has passed four pints and a half of limpid urine. The bowels are soluble.

Ten P.M.—Complains of pain in the back. Since my visit this morning, she has passed four pints of limpid urine.

22d.—Much in the same state as yesterday. She passed three pints of urine from ten o'clock last night to ten this morning. She told me to-day that, for a period of two months before I tapped her, she was in the habit of passing a few table-spoonfuls of urine only in the course of the twenty-four hours, during the whole of that period.

> I prescribed eight drops of the Tincture of Digitalis, to be taken three times a-day; and a dose of the Compound Jalap Powder thrice a-week; and desired her to live on a generous diet.

July 5th.—My attention has not been required since the last report. Her strength has been gradually improving; and to-day she has walked to my house, a distance of a mile from her own. She continued the Digitalis for three days, and then left it off, because it created nausea.

24th.—At four P.M. to-day, I was requested to visit Mrs. Kelly in the capacity of accoucheur, and was informed that the membranes had ruptured. At ten P.M. the child was born, and in ten minutes after the placenta was expelled. The infant was feeble and emaciated: it appeared to be about seven months old, but I thought that it could not survive more than a few days. The dropsical accumulation has again taken place. From the 5th of the month to the present date, this woman has been strong enough to attend to her usual occupation, which is that of a charwoman.

26th.—With the exception of dropsy, Mrs. Kelly is as well as she usually is after an accouchement. On examination, I cannot detect any indurated enlargement of the liver, or of any other viscus. She is thirty-two years of age, has had seven children, and has generally been considered to be a person of strong constitution. My friends, Mr. ANDERSON, of Wilmington-square, and Mr. HENSLEIGH, of Glocester-place, New-road, had seen this patient several times, and were present at the operation.

September 12th.—Mrs. Kelly called upon me to-day, to request that I would tap her once more. She told me that, a fortnight after her accouchement, she began to move about the house, and in a week afterwards to attend to her usual laborious occupation. Her child lived ten days.

22d.—Dyspnœa, cough, quick and hard pulse, render it necessary to let blood ad deliquium animi.

A dose of Calomel and Jalap was ordered.

25th.—The distressing symptoms have increased to an alarming degree, and her abdomen is so large as to render it almost impossible for her to move. A very considerable accumulation of the fluid has taken place since the 12th. I tapped her to-day, and drew off thirty quarts of fluid. During the operation, she was several times threatened with syncope: indeed, we thought it expedient to administer a little gin-and-water, and to allow her to lie down.

26th, two P.M.—All the distressing symptoms are relieved. She passed water three times in the course of the night, about a pint each time.

27th.—Continues as well as she was yesterday.

October 26th.—Circumstances have prevented my paying strict daily attention to this case, since the last report. To-day, she told me that, after I had discontinued my visits, she recovered her strength, and returned to her business; and that nothing particular in her case had since occurred. I perceived that the dropsy was once more forming, and that it would be necessary to tap her again. Necessity obliged her to go to another place of abode, and it is not likely that I shall have an opportunity of seeing her any more.

*Remarks.*—What practical inferences may be deduced from the knowledge and history of cases like this? First, the expediency of the operation; secondly, its safety. Its propriety is advocated by SCARPA, who observes, that "we should puncture the abdomen when ascites, in complication with pregnancy, threatens suffocation." "Cependant," says M. MARC, "si l'hydropsie qui complique la grossesse est assez considerable pour menacer la femme de suffocation, on ne doit pas différer de pratiquer la paracentèse." There are, moreover, several cases on record which corroborate the propriety of the same practice. In the case of Mrs. Kelly, her wretched condition in consequence of her inability to do any office for herself, her anxiety of mind, and the tendency to suffocation, strongly urged the necessity of the operation; and the good effects of it were so speedily obvious as to convince me that the most imminent danger had been prevented. The success of the operation is proved by the fact that a cure has been effected by it.

It appears that, in general, labour commences a short time after the operation. With regard to three of the cases with the history of which I am acquainted, in the first, the woman was delivered the night after she was tapped; in the second, three days after; in the third, seven days after. In the first case, the woman was six months gone with child; in the second, nine months; in the third, six months. The first case recovered, and had no return of ascites. The second case died of hemorrhage; the child lived. The third case recovered, and had no return of ascites; the woman was delivered of dead twins.

24, *Goulden-terrace, Islington; March* 1827.

